# CORO1C expression is associated with poor survival rates in gastric cancer and promotes metastasis *in vitro*

**DOI:** 10.1002/2211-5463.12639

**Published:** 2019-04-29

**Authors:** Xiao Cheng, Xiaonan Wang, Zhengsheng Wu, Sheng Tan, Tao Zhu, Keshuo Ding

**Affiliations:** ^1^ Department of Pathology Anhui Medical University Hefei China; ^2^ Laboratory of Pathogenic Microbiology and Immunology Anhui Medical University Hefei China; ^3^ Hefei National Laboratory for Physical Sciences at Microscale University of Science and Technology of China Hefei China

**Keywords:** CORO1C, Cyclin D1, gastric cancer, Vimentin

## Abstract

Coronin‐like actin‐binding protein 1C (CORO1C) is a member of the WD repeat protein family that regulates actin‐dependent processes by assembling F‐actin. CORO1C was previously reported to promote metastasis in breast cancer and lung squamous cell carcinoma. Here, we investigated the role of CORO1C in gastric cancer. Higher expression levels of CORO1C were detected in gastric cancer tissues as compared with normal gastric tissues. In addition, CORO1C levels were found to be positively correlated with lymph node metastasis in gastric cancer patients. The expression levels of CORO1C were higher in stage III‐IV gastric cancer patients (80.8%) than in stage I‐II gastric cancer patients(57.1%). Gastric cancer patients positive for CORO1C expression showed lower relapse‐free survival and overall survival rates. Knockdown of CORO1C dramatically suppressed total cell number, cell viability, cell colony formation, cell mitosis and cell metastasis, and promoted apoptosis of gastric cancer cells. Furthermore, cyclin D1 and vimentin were found to be positively regulated by CORO1C. As cyclin D1 and vimentin play an oncogenic role in gastric cancer, CORO1C may exert its tumor‐promoting activity through these proteins.

**Abbreviations**
BCL‐2: B‐cell lymphoma 2
CORO1C: Coronin‐like actin‐binding protein 1C
EZH2: Enhancer of zeste homolog 2
GAPDH: glyceraldehyde 3‐phosphate dehydrogenase
IHC: immunohistochemistry
MDM2: mouse double minute 2
MTT: 3‐(4,5‐dimethylthiazol‐2‐yl)‐2,5‐diphenyl‐tetrazolium bromide
OS: overall survival
PBS: phosphate-buffered saline
RFS: relapse‐free survival
RT‐qPCR: reverse transcription quantitative polymerase chain reaction
siNC: negative control siRNA

Gastric cancer is the fifth most common cancer worldwide and ranks third among cancer‐related deaths in the world 1. Although the incidence of gastric cancer has been decreasing in recent years, gastric cancer is still one of the most common types of cancer in East Asian countries, especially in China 2. A combination of surgery, chemotherapy and radiotherapy is the best choice for gastric cancer therapy. Although therapeutic methods for human cancers are constantly improving, the recurrence rate of gastric cancer is still high, the prognosis of gastric cancer patients is poor, and the mean 5‐year survival rate is no more than 20% 2. The mechanisms involved in tumor initiation and development of gastric cancer have been studied for many years, but the underlying mechanisms are still poorly understood. Further study of the intrinsic mechanisms involved in gastric cancer and development of new effective methods of therapy are needed.

Coronin‐like actin‐binding protein 1C (CORO1C), also known as HCRNN4, is a member of the WD repeat protein family whose members contain a 40‐amino‐acid minimum conserved region generally enclosed by Gly‐His and Trp‐Asp. It was reported to regulate actin‐dependent processes by assembling F‐actin 3. As reported previously, CORO1C promotes cellular metastasis in human breast cancer 4 and lung‐squamous cell carcinoma 5. CORO1C promoted invasion of breast cancer and glioma cells by specifically promoting the formation of invadopodia 6, 7. High levels of CORO1C were associated with poor prognosis in human hepatocellular carcinoma patients 8 and primary effusion lymphoma patients 9. These studies suggested that CORO1C possessed an oncogenic role in some kinds of human cancers. However, there is no report about the relationship between CORO1C and gastric cancer. The effect of CORO1C on human gastric cancer is still indistinct.

Herein, we examined whether CORO1C was oncogenic for human gastric cancer. As determined by immunohistochemistry, the protein levels of CORO1C were much higher in gastric cancer tissues than in normal gastric tissues. High expression levels of CORO1C were positively correlated with poor clinicopathological parameters in gastric cancer patients, including lymph node metastasis and clinical stage. Moreover, CORO1C expression levels in gastric cancer patients were negatively correlated with both overall survival (OS) rate and relapse‐free survival (RFS) rate. As determined by cell functional assays, knocking down of *CORO1C* in gastric cancer cells significantly decreased cellular ability for growth and metastasis. Knocking down of *CORO1C* in gastric cancer cells notably promoted cellular apoptosis and inhibited the process of cell cycle. Cyclin D1 (a member of the highly conserved cyclin family that has a role in promoting cell cycle progression 10) and vimentin (a type III intermediate filament protein that has an important role in maintaining the integrity of cytoplasm and the shape of cells, and is involved in cellular migration, attachment and signaling 11) were positively regulated by CORO1C, as determined by reverse transcription quantitative PCR (RT‐qPCR). Cyclin D1 and vimentin might mediate the oncogenicity in human gastric cancer caused by CORO1C. Therefore, CORO1C possessed a tumor promoting role in human gastric cancer. CORO1C could be used as a potential target for gastric cancer diagnosis and treatment.

## Materials and methods

### Clinical samples

Eighty human gastric cancer tissues and 80 normal human gastric tissues embedded with paraffin were gathered from the Department of Pathology, First Affiliated Hospital of Anhui Medical University (Hefei, Anhui, China). These gastric cancer and normal gastric tissues were from patients who had undergone resection in the First Affiliated Hospital of Anhui Medical University between 2012 and 2013. Patients with other diseases or other related surgical history were excluded. They were followed up through telephone call for at least 5 years, and their survival rates were documented. We obtained informed consent from every patient involved in this study before we performed the work. This research was authorized by the Institutional Review Boards of Anhui Medical University and was performed on the basis of The Code of Ethics of the World Medical Association (Declaration of Helsinki).

### Immunohistochemistry

CORO1C protein expression levels of these 160 sections of gastric tissues were determined by immunohistochemistry (IHC) analysis, using the Ultra Sensitive‐SP kit (Maixin‐Bio, Fuzhou, China), essentially as performed earlier 12, 13. CORO1C rabbit polyclonal antibody (Proteintech Group, Inc., Chicago, IL, USA, 1 : 100) was used. The stained sections were evaluated independently by two senior pathologists. Sections with ≥ 10% stained cells were defined as CORO1C positive, and sections with < 10% stained cells were defined as CORO1C negative.

### Cell culture

Human gastric cancer cell lines BGC‐823 and AGS [obtained from ATCC (the American Type Culture Collection) Rockville, MD, USA] were used in this study. Both cell lines were grown in RPMI 1640 medium (Invitrogen, Waltham, MA, USA) containing 10% FBS (Invitrogen) and were cultured in a humidified atmosphere with 5% CO_2_ at 37 °C as recommended.

### siRNA transfection

siRNAs [including si*CORO1C‐1*, si*CORO1C‐2* and si*NC* (negative control siRNA)] were synthesized by GenePharma (Shanghai, China). As described previously 14, 15, all siRNAs were transfected using Lipofectamine 2000 (Invitrogen) as recommended by the manufacturer. Specific siRNA sequences were as follows: si*CORO1C‐1*, 5′‐GGCAAUAACAGCUGGGCUATT‐3′; si*CORO1C‐2*, 5′‐GCAUCCAACGGCCCGCAAUTT‐3′; and si*NC*, 5′‐UUCUCCGAAC GUGUCACGUTT‐3′.

### Western blot

Protein levels of CORO1C, cyclin D1 and vimentin were determined by western blot essentially as described earlier 12, 13, 16. CORO1C rabbit polyclonal antibody, cyclin D1 rabbit polyclonal antibody, vimentin rabbit polyclonal antibody (all 1 : 1000; Proteintech Group), and mouse monoclonal antibody against β‐actin (1 : 5000; Sigma‐Aldrich, St. Louis, MO, USA) were used.

### Cell functional assays

Cell functional assays, including total cell number assay, 3‐(4,5‐dimethylthiazol‐2‐yl)‐2,5‐diphenyl‐tetrazolium bromide (MTT) assay, and cell colony formation, migration and invasion experiments were carried out as performed previously 12, 13, 14, 15. In total cell number assay, 1 × 10^5^ original cells were seeded into six‐well plates and cultured in normal conditions, and then these cells were counted every day for 5 days. In the MTT assay, 2000 original cells were seeded into 96‐well plates and cell viability (absorbance at 570 nm) was tested after 72 h. In detail, after discarding the cell culture medium (RPMI 1640 medium containing 10% FBS), 1 : 9 MTT mixture [mixed with 100 μL 5 mg·mL^−1^ MTT (A100793; Sangon Biotech, Shanghai, China) and 900 μL cell culture medium] was added into the 96‐well plates (100 μL per well). The cells were cultured in an incubator (5% CO_2_, 37 °C) for 2 h and then the MTT mixture in 96‐well plates was changed for DMSO (200 μL per well). After mixing away from light for 15 min, the absorbance at 570 nm was detected. In the cell colony formation assay, 1000 original cells were seeded into six‐well plates and cell colony formation was detected after 10 days. In detail, after discarding the cell culture medium (RPMI 1640 medium containing 10% FBS), the cells in the six‐well plates were washed with phosphate‐buffered saline (PBS) and then fixed with 4% formaldehyde (200 μL per well). Cells in six‐well plates were washed with PBS and then the cells were dyed with 0.1% crystal violet (A100528; Sangon Biotech; 100 μL per well) at room temperature. After incubating for 10 min, the cells were washed with PBS again and the cell colony numbers were counted and images were collected with an Olympus microscope (Olympus, Tokyo, Japan). For the cell migration assay, cells were directly seeded into transwells and were detected after 24 h. In detail, after cells were counted, the mixture of cells and RPMI 1640 medium with no FBS was added into the upper chambers (1 × 10^5^ cells per well). RPMI 1640 medium containing 5% FBS was added to the lower chambers. Both of BGC‐823 and AGS cells were cultured in an incubator (5% CO_2_, 37 °C) and detected after 24 h. For the cell invasion assay, cells were seeded into transwells with Matri‐gel (BD Biosciences, San Jose, CA, USA) and were detected after 48 h. In detail, after cells were counted, the mixture of cells and RPMI 1640 medium with no FBS was added into the upper chambers coated with Matri‐gel (1 × 10^5^ cells per well). RPMI 1640 medium containing 10% FBS was added to the lower chambers. Both BGC‐823 and AGS cells were cultured in an incubator (5% CO_2_, 37 °C) and detected after 48 h. After that, the lower chambers were discarded and the upper chambers (for both migration and invasion assay) were put into chambers with PBS and washed for twice. Then the cells in the upper chambers were dyed with 0.1% crystal violet (A100528; Sangon Biotech) for 10 min at room temperature and then washed with PBS three times. The Matri‐gel was erased with a swab, and finally, the cell numbers were counted. Images were collected with an Olympus microscope (Olympus) and the migrated or invaded cells were counted.

### Flow cytometry

Cellular apoptosis and cell cycle analysis of BGC‐823 and AGS cells were determined by flow cytometry. After transfection with siRNAs for 72 h, BGC‐823 and AGS cells were gathered and washed with cold PBS twice. Then, the cells were suspended by 400 μL incubation solution (the cell concentration was approximately 1 × 10^6^ cells·mL^−1^). For cell apoptosis analysis, cells were incubated with annexin V–FITC (Beyotime, Shanghai, China) and propidium iodide (Beyotime) at 4 °C away from the light for 0.5 h. Cellular apoptosis was examined by flow cytometric analysis. For cell cycle analysis, cells were incubated with propidium iodide and Rnase A for 0.5 h, and flow cytometric analyis was carried out.

### Reverse transcription quantitative PCR

The mRNA levels of enhancer of zeste homolog 2 (*EZH2*), B‐cell lymphoma 2 (*BCL‐2*), *cyclin D1*, mouse double minute 2 (*MDM2*), *vimentin*, and *C‐myc* were measured by RT‐qPCR, which was carried out as performed previously 14, 15. Glyceraldehyde 3‐phosphate dehydrogenase (*GAPDH*) was selected as internal reference gene. The primer sequences used were: *EZH2*, forward 5′‐GCTTCCCAATAACAGTAGCAGG‐3′ and reverse 5′‐TTCAGCACCACTCCACTCCAC‐3′; *BCL‐2*, forward 5′‐GACTTCGCCGA GATGTCCAG‐3′ and reverse 5′‐CTCAAA GAAGGCCACAATCCTC‐3′; *cyclin D1*, forward 5′‐CTGGTGAACAAGCTCAAGTGG‐3′ and reverse 5′‐GAGGCGGT AGTAGGACAGGAAG‐3′; *MDM2*, forward 5′‐TGAATCTACAGGGACGCCA TC‐3′ and reverse 5′‐CCTGATCCAACCAATCACCTG‐3′; *vimentin*, forward 5′‐CACCAACGAGAAGGTGGAGC‐3′ and reverse 5′‐TGGTTAGCTGGTCCA CCTGC‐3′; *C‐myc*, forward 5′‐AGACTCCAGCGCCTTCTCTC‐3′ and reverse 5′‐GCACCTCTTGAGGACCAGTG‐3′; *GAPDH*, forward 5′‐TGCACCACCAA CTGCTTAGC‐3′ and reverse 5′‐GGCATGGACTGTGGTCATGAG‐3′.

### Statistical analysis

In this study, every test was repeated at least three times and the results represented the final average. For immunohistochemistry and clinicopathological parameter‐related analysis, the chi‐square test was used. For RFS and OS rate analysis of patients, Kaplan–Meier curves were used. For cell total number analysis, MTT assay, cell colony formation, flow cytometry, cell migration, cell invasion experiments and RT‐qPCR, Student's unpaired two‐tailed *t* test was used. *P* < 0.05 was considered statistically significant.

## Results

### Expression of CORO1C in human gastric cancer tissues and normal gastric tissues

**Figure 1:**
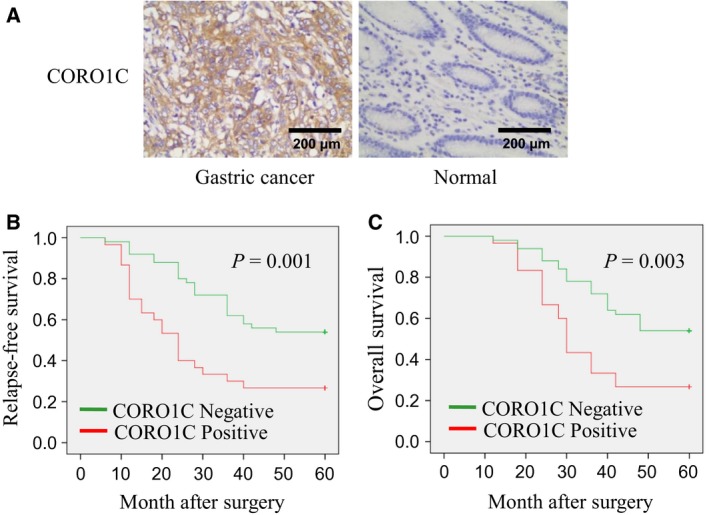
Expression of CORO1C in gastric cancer tissues and normal gastric tissues; association between CORO1C expression and patients' survival rates. (A) Protein levels of CORO1C in gastric cancer tissues and normal gastric tissues were detected using immunohistochemistry. Scale bar, 200 μm. (B,C) Kaplan–Meier curves showing the RFS and OS rates of gastric cancer patients with different CORO1C expression levels. Sections with ≥ 10% stained cells were defined as CORO1C positive, and sections with < 10% stained cells were defined as CORO1C negative.

Immunohistochemistry was performed to detect the protein levels of CORO1C in 80 human gastric cancer tissues and 80 normal gastric tissues. As illustrated in Fig. 1A, immunoreactive CORO1C protein was mostly distributed in the cytoplasm. Thirty‐two out of 80 normal gastric tissues showed positive expression of CORO1C (40.0%), and 48 out of 80 cases showed negative expression of CORO1C (60.0%). On the contrary, 58 out of 80 gastric cancer tissues showed positive expression of CORO1C (72.5%), and 22 out of 80 cases showed negative expression of CORO1C (27.5%) (*P* < 0.001) (Table 1). As a result, CORO1C protein levels in gastric cancer tissues were higher compared with normal gastric tissues.

**Table 1:** Expression of CORO1C in gastric cancer and normal gastric tissues.

Group	*n*	CORO1C expression	
		Negative, *n* (%)	Positive, *n* (%)
Gastric cancer	80	22 (27.5)	58 (72.5)*
Normal	80	48 (60.0)	32 (40.0)

*P < 0.001. χ^2 ^= 17.168.

### Correlations between CORO1C expression and clinicopathological parameters/survival rates in patients with gastric cancer

We analyzed the correlation between CORO1C expression and clinicopathological parameters in these 80 gastric cancer patients. Patient gender, age, tumor size, lymph node metastasis, clinical stage and histological grade were included. As illustrated in Table 2, gastric cancer patients with positive CORO1C expression exhibited a higher risk of lymph node metastasis (*P *=* *0.005) and a worse clinical stage (*P *=* *0.024) compared with patients with negative CORO1C expression. However, the correlations between CORO1C expression and other clinicopathological parameters were not statistically significant (all *P* > 0.05).

**Table 2:** Association of CORO1C expression with clinicopathological parameters from gastric cancer patients.

Parameter	*n*	CORO1C expression (*n* (%))		*P*	χ^2^
		Negative	Positive		
Age (years)					
≤ 60	38	11 (28.9)	27 (71.1)	0.783	0.076
> 60	42	11 (26.2)	31 (73.8)		
Gender					
Male	44	12 (27.3)	32 (72.7)	0.960	0.003
Female	36	10 (27.8)	26 (72.2)		
Tumor size (cm)					
≤ 5	48	14 (29.2)	34 (70.8)	0.683	0.167
> 5	32	8 (25.0)	24 (75.0)		
Lymph node metastasis					
No	28	13 (46.4)	15 (53.6)	0.005	7.741
Yes	52	9 (17.3)	43 (82.7)		
Grade					
I–II	47	13 (27.7)	34 (72.3)	0.970	0.001
III	33	9 (27.3)	24 (72.7)		
Stage					
I–II	28	12 (42.9)	16 (57.1)	0.024	5.096
III–IV	52	10 (19.2)	42 (80.8)		

For further study, these gastric cancer patients were followed up for more than 5 years, and their RFS and OS rates were calculated. As illustrated in Fig. 1B,C, gastric cancer patients with positive CORO1C expression showed significantly lower RFS (*P *=* *0.001) and OS (*P *=* *0.003). Therefore, the positive expression of CORO1C indicated poor prognosis in patients with gastric cancer.

### CORO1C promoted cell proliferation in gastric cancer cells

**Figure 2:**
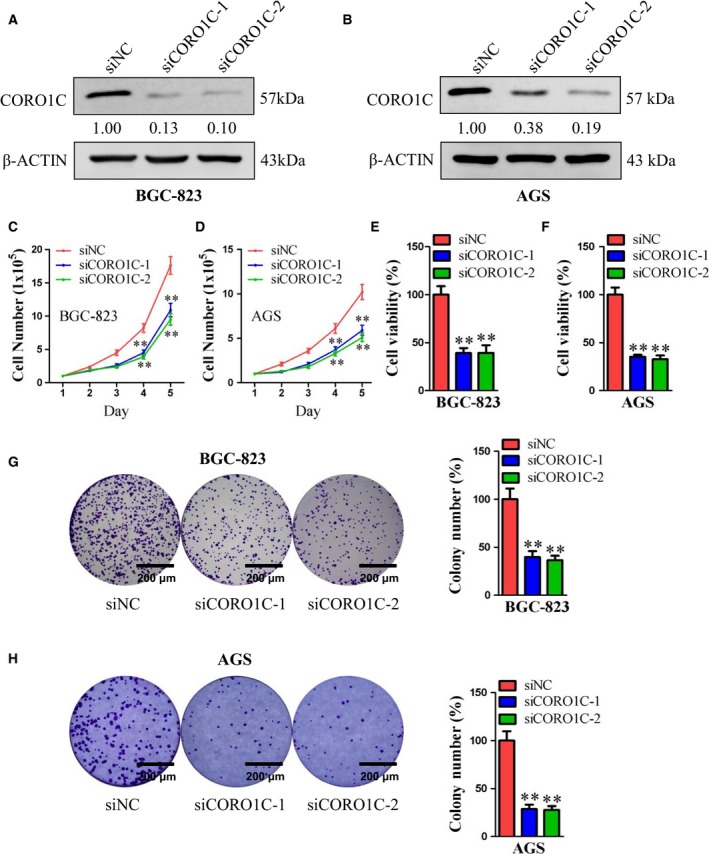
CORO1C promoted cell proliferation of human gastric cancer cells. BGC‐823 and AGS cells were transfected with si*CORO1C‐1*, si*CORO1C‐2* or si*NC*. (A,B) Protein levels of CORO1C were detected by western blot. β‐Actin was used as control. (C,D) Cell total number assay was performed with an original cell number of 1 × 10^5^. Cells were counted every day for 5 days. (E,F) MTT assay was performed to evaluate cell viability (BGC‐823‐si*NC* 100.00%; BGC‐823‐si*CORO1C‐1* 39.18%, BGC‐823‐si*CORO1C‐2* 39.30%, both *P* < 0.01 *vs* si*NC*; AGS‐si*NC* 100.00%; AGS‐si*CORO1C‐1* 35.31%, AGS‐si*CORO1C‐2* 32.67%, both *P* < 0.01 *vs* si*NC*). (G,H) Cell colony formation assay was performed with an original cell number of 1000, and cell colony numbers were calculated after 10 days (BGC‐823‐si*NC* 100.00%; BGC‐823‐si*CORO1C‐1* 39.84%, BGC‐823‐ si*CORO1C‐2* 36.54%, both *P* < 0.01 *vs* si*NC*; AGS‐si*NC* 100.00%; AGS‐si*CORO1C‐1* 28.68%, AGS‐si*CORO1C‐2* 27.67%, both *P* < 0.01 *vs* si*NC*). Scale bar, 200 μm. All data are shown as the mean ± standard deviation and were evaluated using unpaired two‐tailed *t* test. Every test was repeated at least three times and the results represented the average (*n* ≥ 3). **P <* *0.01.

Gastric cancer cell lines BGC‐23 and AGS were selected in this research for cell functional experiments. As illustrated in Fig. 2A,B, after transfection with si*CORO1C‐1* or si*CORO1C‐2*, the protein levels of CORO1C decreased significantly in both AGS and BGC‐23 cells. In monolayer culture, si*CORO1C‐1* and si*CORO1C‐2* dramatically reduced total cell number of both BGC‐823 cells and AGS cells during a period of 5 days compared with si*NC* (Fig. 2C,D). Concordantly, after transfection with si*CORO1C‐1* or si*CORO1C‐2*, cell viability of both BGC‐823 cells and AGS cells significantly decreased compared with si*NC* as measured by MTT assay (Fig. 2E,F). Cellular colony formation was also obviously reduced after knocking down CORO1C by si*CORO1C‐1* or si*CORO1C‐2* in both BGC‐823 and AGS cells (Fig. 2G,H). Therefore, CORO1C played a promoting role in cellular proliferation of gastric cancer cells.

### CORO1C inhibited cellular apoptosis and facilitated cell mitosis in gastric cancer cells

**Figure 3:**
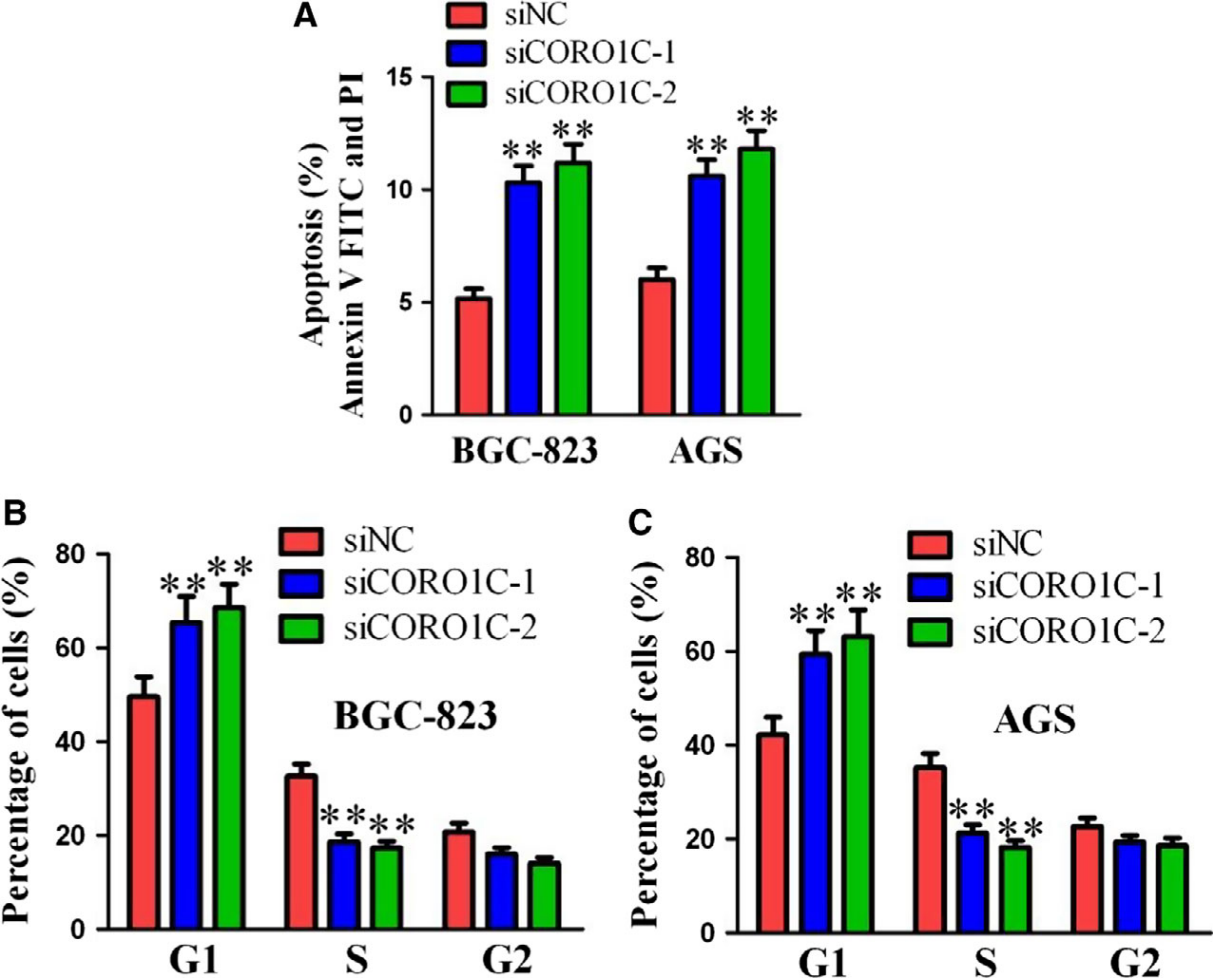
CORO1C inhibited cellular apoptosis and facilitated cell mitosis of human gastric cancer cells. BGC‐823 and AGS cells were transfected with si*CORO1C‐1*, si*CORO1C‐2* or si*NC*. (A) Cellular apoptosis analysis was performed by flow cytometry. (B,C) Cell cycle analysis was performed by flow cytometry. All data are shown as the mean ± standard deviation and were evaluated using unpaired two‐tailed *t* test. Every test was repeated at least three times and the results represented the average (*n* ≥ 3). **P <* *0.01.

Flow cytometry was performed to explore the effects of CORO1C on cellular apoptosis and the cell cycle in gastric cancer cells. As illustrated in Fig. 3A, after transfection with si*CORO1C‐1* or si*CORO1C‐2*, the percentage of cellular apoptosis increased notably in both BGC‐823 and AGS cells compared with si*NC*. For cell cycle analysis, the percentage of G1 phase cells increased notably and the percentage of S phase cells decreased notably after knocking down of CORO1C by si*CORO1C‐1* or si*CORO1C‐2* compared with si*NC* in both BGC‐823 and AGS cells (Fig. 3B,C). Therefore, CORO1C inhibited cellular apoptosis and facilitated cell mitosis in gastric cancer cells.

### CORO1C promoted cellular metastasis in gastric cancer cells

**Figure 4:**
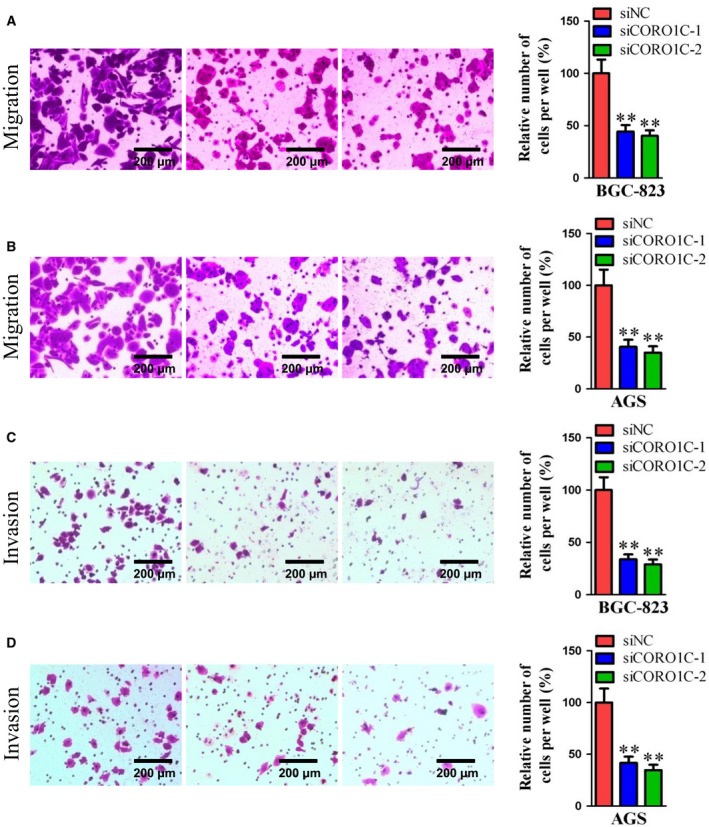
CORO1C promoted cell migration and invasion of human gastric cancer cells. BGC‐823 and AGS cells were transfected with si*CORO1C‐1*, si*CORO1C‐2* or si*NC*. (A,B) Cell migration assay was performed; 1 × 10^5^ cells were seeded in transwells and cell migration was detected after 24 h in both BGC‐823 and AGS cells (BGC‐823‐si*NC* 100.00%; BGC‐823‐si*CORO1C‐1* 44.26%, BGC‐823‐si*CORO1C‐2* 40.24%, both *P* < 0.01 *vs* si*NC*; AGS‐si*NC* 100.00%; AGS‐si*CORO1C‐1* 40.60%, AGS‐si*CORO1C‐2* 34.90%, both *P* < 0.01 *vs* si*NC*). Scale bar, 200 μm. (C,D) Cell invasion assay was performed; 1 × 10^5^ cells were seeded in transwells with Matri‐gel and cell invasion was detected after 48 h in both BGC‐823 and AGS cells (BGC‐823‐si*NC* 100.00%; BGC‐823‐si*CORO1C‐1* 33.69%, BGC‐823‐ si*CORO1C‐2* 28.96%, both *P* < 0.01 *vs* si*NC*; AGS‐si*NC* 100.00%; AGS‐si*CORO1C‐1* 41.63%, AGS‐si*CORO1C‐2* 34.69%, both *P* < 0.01 *vs* si*NC*). Scale bar, 200 μm. All data are shown as the mean ± standard deviation and were evaluated using unpaired two‐tailed *t* test. Every test was repeated at least three times and the results represented the average (*n* ≥ 3). **P <* *0.01.

For further study, cellular migration and invasion experiments were carried out to assess the functional role of CORO1C in cellular migration and invasion of gastric cancer cells. As illustrated in Fig. 4A–D, both cellular migration and cellular invasion decreased notably after knocking down CORO1C by si*CORO1C‐1* or si*CORO1C‐2* compared with si*NC* in both gastric cancer cell lines BGC‐823 and AGS. Therefore, CORO1C also performed a promoting role in cellular metastasis of human gastric cancer cells.

### CORO1C promoted the expression of cyclin D1 and vimentin in human gastric cancer cells

**Figure 5:**
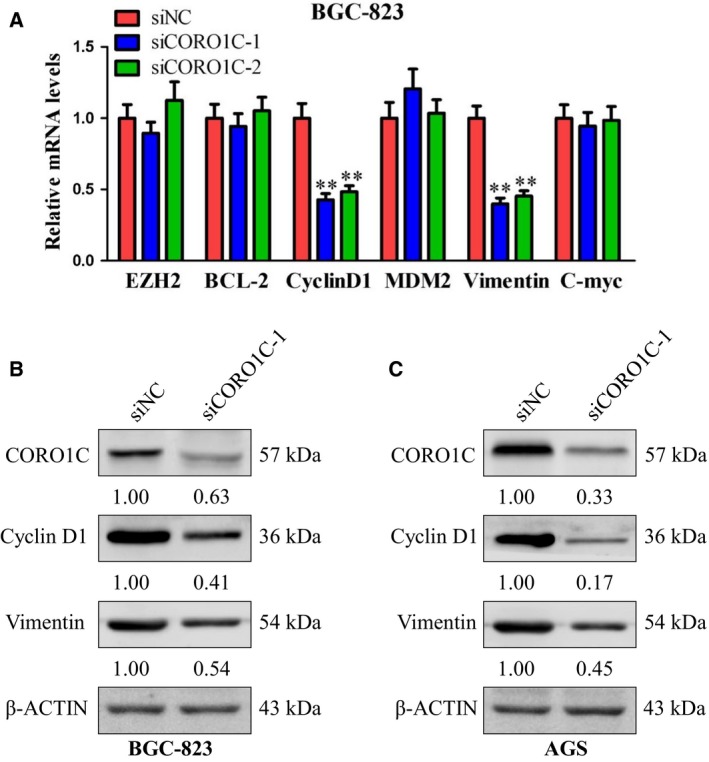
CORO1C promoted the expression of cyclin D1 and vimentin in gastric cancer cells. (A) mRNA levels of *EZH2*, *BCL‐2*, *cyclin D1*, *MDM2*, *vimentin* and *C‐myc* in BGC‐823 cells after transfected with si*CORO1C‐1*, si*CORO1C‐2* or si*NC* were detected using RT‐qPCR. (B,C) BGC‐823 and AGS cells were transfected with si*CORO1C‐1* or si*NC*. Protein levels of CORO1C, cyclin D1 and vimentin were detected using western blot. β‐Actin was detected as control. All data are shown as the mean ± standard deviation and were evaluated using unpaired two‐tailed *t* test. Every test was repeated at least three times and the results represented the average (*n* ≥ 3). **P <* *0.01.

To unveil the downstream genes involved in the tumor promoting role of CORO1C in human gastric cancer cells, several candidate genes (including *EZH2*, *BCL‐2*, *cyclin D1*, *MDM2*, *vimentin* and *C‐myc*) were selected according to the literature and our former work (they were oncogenes in gastric cancer and might be regulated by CORO1C), and we firstly examined the mRNA levels of these genes by RT‐qPCR after transfection with si*CORO1C‐1* or si*CORO1C‐2* in BGC‐823 cells. As illustrated in Fig. 5A, mRNA levels of *cyclin D1* and *vimentin* decreased notably in BGC‐823‐si*CORO1C‐1* and BGC‐823‐si*CORO1C‐2* cells compared with control cells. However, no significant changes of *EZH2*, *BCL‐2*, *MDM2* or *C‐myc* mRNA levels were observed in either BGC‐823‐si*CORO1C‐1* or BGC‐823‐si*CORO1C‐2* cells. To confirm this result, the protein levels of cyclin D1 and vimentin in BGC‐823‐si*CORO1C‐1* and AGS‐si*CORO1C‐1* cells were examined by western blot. Concordant with former results, CORO1C protein levels decreased notably in BGC‐823‐si*CORO1C‐1* and AGS‐si*CORO1C‐1* cells compared with si*NC* cells. Moreover, the protein expression levels of both cyclin D1 and vimentin also decreased dramatically in BGC‐823‐si*CORO1C‐1* and AGS‐si*CORO1C‐1* cells compared with control (Fig. 5B,C). As reported previously, *cyclin D1* and *vimentin* were important genes contributing to tumor growth, cell proliferation, migration and invasion of gastric cancer 17, 18, 19, 20, 21. Therefore, *cyclin D1* and *vimentin* might mediate the process of CORO1C increasing oncogenicity in human gastric cancer cells.

## Discussion

The mechanisms of initiation and progression of human gastric cancer have always been a tough problem for medical researchers. In this study, we systematically investigated the functional role of CORO1C in human gastric cancer. Compared with normal gastric tissues (40%), notably higher expression levels of CORO1C were detected in gastric cancer tissues (72.5%), suggesting that the occurrence of gastric cancer may be related to the high expression of CORO1C. CORO1C expression levels were positively related with lymphatic metastasis (82.7%), and higher clinical stage gastric cancer patients (80.8%) tended to express more CORO1C than lower clinical stage gastric cancer patients (57.1%), showing that poor clinicopathological parameters, including lymph node metastasis and clinical stage, were associated with the high levels of CORO1C in gastric cancer patients. Gastric cancer patients with positive expression of CORO1C showed both lower RFS rate and lower OS rate compared with patients with negative expression of CORO1C. Therefore, positive expression of CORO1C indicated poor prognosis in patients with gastric cancer. Moreover, total cell number (examined by total cell number assay), cell viability (examined by MTT assay), cell colony formation (examined by cell colony formation assay, cell migration (examined by cell migration assay) and cell invasion (examined by cell invasion assay) all decreased significantly after knocking down of CORO1C by the siRNA method, indicating that CORO1C promoted cellular proliferation and metastasis in gastric cancer cells. The percentage of apoptosis increased, the proportion of cells in G1 phase increased and in S phase decreased significantly after depletion of CORO1C in gastric cancer cells, indicating that CORO1C depressed cell apoptosis and stimulated cellular mitosis in gastric cancer cells. Therefore, CORO1C is oncogenic for human gastric cancer. As reported previously, CORO1C acted as a pivotal regulator of actin activity participating in the assembly of the actin‐related protein 2/3 (Arp 2/3) complex and the disintegration of cofilin 22. CORO1C participated in the carcinogenicity of hepatocellular cancer by activating Rac‐1 23. As a direct target of the miR‐*1/133a* cluster, CORO1C played a promoting role in cellular proliferation, invasion and migration of lung‐squamous cell cancer 5. Hepatocellular carcinoma patients with positive expression of CORO1C showed a worse clinical stage compared with patients with negative CORO1C expression 8. Knocking down of CORO1C inhibited metastasis of human breast cancer cells 4. These results all support our present data. Therefore, CORO1C was oncogenic in several kinds of human cancers, and here we have first proved the oncogenic role of CORO1C in human gastric cancer.

For the downstream mechanisms, cyclin D1 and vimentin were detected to be positively regulated by CORO1C. As reported previously, cyclin D1 was detected to mediate cellular cycle change and proliferation guided by WDR5 in gastric cancer 24. Cyclin D1 was involved in the AKT–glycogen synthase kinase 3 β–β‐catenin–cyclin D1 signaling pathway that participated in the process of extracellular 5′‐nucleotidase (CD73) promotion of cell growth of human breast cancer 25. Regulated directly by miR‐*720*, cyclin D1 played a promoting role in both cellular proliferation and metastasis in pancreatic cancer 26. Activation of cyclin D1 enhanced proliferation of human colorectal cancer cells 27. In addition, cyclin D1 was also identified to be oncogenic for human urinary bladder cancer, liver cancer, non‐small cell lung cancer, cervical cancer, etc. 28, 29, 30, 31, 32 As for vimentin, it was reported that its protein levels could be suppressed by miR‐*320a* or miR‐*373*, consequently inhibiting tumor growth, cellular proliferation and metastasis in gastric cancer cells 29, 32. Vimentin was determined to acted as a poor prognostic factor in patients with gastric cancer 33. Vimentin has also been detected as an effective clinical treatment target for malignant pleural mesothelioma 34. Furthermore, vimentin was reported to play oncogenic roles in many other human cancers, including nasopharyngeal carcinoma, tongue squamous cell carcinoma, ovarian cancer, hepatocellular cancer and pancreatic cancer 35, 36, 37, 38, 39. In our present study, we have determined that CORO1C positively regulated the expression of cyclin D1 and vimentin. Therefore, the promoting role of CORO1C in both proliferation and metastasis of gastric cancer cells might be mediated by cyclin D1 and vimentin.

As reported previously, stage IIIb–IV gastric cardia adenocarcinoma patients tended to express more cyclin D1 compared with stage I–IIIa gastric cardia adenocarcinoma patients 40. Vimentin levels were positively correlated with lymph node metastasis and TNM stage in gastric cancer patients 33. Therefore, high levels of cyclin D1 or vimentin indicated poor prognosis in gastric cancer patients. According to our findings, cyclin D1 and vimentin were positively regulated by CORO1C. The positive expression of CORO1C also indicated poor prognosis in patients with gastric cancer. Therefore the CORO1C–cyclin D1–vimentin pathway is in turn associated with the prognosis of gastric cancer patients. According to the literature, CORO1C participated in the tumorigenicity of hepatocellular cancer by activating Rac‐1 23. Rac‐1 was reported to promote cellular proliferation of human colon cancer by up‐regulating the Jun N‐terminal kinase 2–C‐Jun–cyclin D1 pathway 41 and promote mitochondrial motion by causing the phosphorylation of vimentin 42, which suggested that Rac‐1 might provoke cellular proliferation and metastasis by regulating cyclin D1 and vimentin. The CORO1C–Rac‐1–cyclin D1–vimentin pathway might also contribute to human gastric cancer and mediate the enhanced carcinogenicity of gastric cancer cells, which needs to be examined in future work.

In summary, we have demonstrated that CORO1C promoted both proliferation and metastasis, stimulated cellular mitosis and inhibited cell apoptosis in gastric cancer cells. Gastric cancer patients with positive CORO1C expression were associated with poor prognosis and lower survival rate. Cyclin D1 and vimentin were positively regulated by CORO1C. The tumor promoting role of CORO1C might be mediated by cyclin D1 and vimentin. Therefore, CORO1C was a carcinogenic factor in gastric cancer, and it could serve as a potential target for gastric cancer diagnosis and therapy.

## Conflict of interest

The authors declare no conflict of interest.

## Author contributions

XC performed cell functional study and tissue study, and participated in the design of the study. XW performed cell functional experiments and mRNA analysis. ZW performed cell functional experiments and western blot. ST participated in tissue experiments and data analysis. TZ participated in manuscript revision. KD designed the study and wrote the manuscript.
